# Bio-evaluation of functional date bars using rats as model organism against hypercholesterolemia

**DOI:** 10.1186/s12944-019-1087-3

**Published:** 2019-07-04

**Authors:** Muhammad Sajid Arshad, Syeda Mamoona Batool, Muhammad Kamran Khan, Muhammad Imran, Muhammad Haseeb Ahmad, Faqir Muhammad Anjum, Shahzad Hussain

**Affiliations:** 1 Institute of Home and Food Sciences, Government College University, Faisalabad, Pakistan; 2 Vice Chancellor Secretariat, University of the Gambia, Banjul, The Gambia; 3 College of Food and Agricultural Sciences, King Saud University, Riyadh, Saudi Arabia

**Keywords:** Date varieties, Rats modelling, Antioxidant profile, Hypercholesterolemia

## Abstract

**Background:**

The present research project was designed to evaluate the cholesterol lowering potential of different date varieties including one exotic (Ajwa) and three Pakistani varieties (Aseel, Khudravi, Hallawi).

**Methods:**

The albino rats were divided into six groups on the basis of different diets which includes, control having basal diet, high cholesterol high sucrose diet, high cholesterol high sucrose diet plus Khudravi dates, high cholesterol high sucrose diet plus Hallawi dates, high cholesterol high sucrose diet plus Aseel dates, high cholesterol high sucrose diet plus Ajwa dates to evaluate maximum cholesterol lowering potential of each date variety.

**Results:**

The results showed that Hallawi and Ajwa have lower crude fiber content as 2.02 ± 0.03% and 2.43 ± 0.04% however, lowest crude fat content (0.26 ± 0.01%) was also observed in ajwa. Mineral profile depicted that sodium (9.50–18.00 mg/100 g) was found to be in lesser amount among all varieties whereas, higher amount of potassium (465.00 to 887.20 mg/100 g) depicted that it is suitable for people having hypertension. Higher amount of reducing sugar was also observed in ajwa (79.45 ± 1.22%) followed by Hallawi (77.68 ± 1.42%). Total phenolic contents were found higher in Aseel (291.36 mg/100 g) whereas, minimum was observed in Khudravi (232.64 mg/100 g). Furthermore, date varieties were also examined rat modeling to evaluate their maximum cholesterol lowering efficiency. Ajwa and Hallawi were observed to suppress the cholesterol efficiently as 110 mg/dL and 103 mg/dL respectively. On the basis of chemical profiling and other parameters, two date varieties Ajwa and Hallawi showed almost similar results and found to have maximum serum cholesterol, LDL and triglyceride reduction potential with good kidney and liver functions. Functional date bar was also developed by using Hallawi variety andsubjected to sensory evaluation.

**Conclusion:**

In nutshell, Hallawi date variety was considered as better cholesterol lowering potential among other indigenous varieties and very close to Ajwa variety. So that Hallawi can be used to suppress the deadly effects of obesity and allied discrepancies particularly hypercholesterolemia.

## Introduction

The diseases which are associated with life style are due to the behavioral and dietary risk factors which have hazardous effects on metabolic and physiological conditions of people. According to World health organization (WHO), these diseases are also called the non-communicable diseases (NCD) and death burden was 57 million out of which 36 million were due to NCDs worldwide [1]. In low and middle income countries death rate are due to NCDs and 80% contributing major and frequent cause of adult mortality and morbidity globally [2]. Major types of non-communicable diseases are CVDs, cancer, diabetes, obesity, neurological disorders and respiratory diseases. From past few decades CVDs remain the single largest cause of death representing 30% of all deaths and about 50% of NCDs deaths [3, 4]. Several modifiable and non-modifiable risk factors are associated with the occurrence of non-communicable metabolic diseases. World health organization (WHO) has prioritized modifiable risk factors including unhealthy diet, less physical activity and metabolic abnormalities like serum lipid disorder to prevent or reduce the occurrence of diseases. Elevation of metabolic risks is directly linked with food we consume and how we utilize that food energy. These foods only provide fats and sugars which only exert unhealthy effects on human health. Consumption of energy dense foods and less physical activities are becoming a leading cause of obesity and other metabolic factors like hypercholesterolemia, hypertension, insulin resistance and many others. Interventions supported by dietary recommendations pose healthier effects in lowering metabolic risks [5]. Diet based therapy includes use of organic foods such as fruits and vegetables due to the presence of phytochemicals (dietary fiber, carotenoids and polyphenols (phenolics and flavonoids) in them which have pharmacological potential against diseases. Date fruit is very much popular among the Muslim community. Pakistan’s commercially important date varieties include Begum Jangi, Aseel, Fasli, Muzawati, Hillawi, Khudravi, Rabai and Dhakki. Global date production was 6.9 million tons and Pakistan is among top ten date producing countries and during the year 2007–08 production of date palm (*Phoenix dactylifera*) in Pakistan was 557.6 thousand [6]. Dates have both nutritious and medicinal benefits and are called “Tree of life” by Arabs. Higher concentration of polyphenols is present in dried fruits and dates with admirable nutritional profile that enhance the lipoproteins in blood and prevent from oxidation [7]. Date fruit contain carbohydrates (44–48%), dietary fiber (9.6%, with 2.5%), minute amount of fat (0.2 to 0.5%), protein (2.3 to 5.6%) and higher amount of macro minerals potassium calcium and vitamins. Its composition makes it suitable to lower the occurrence of certain coronary diseases, diabetes and most important obesity which is a major heath threatening risk factor [8]. It is the need of time to produce foods with high nutritional quality that could be useful in providing the necessary nourishment and combating malnutrition and nutrient deficiencies, in this regard development of functional foods containing valuable phytochemicals is of great attention worldwide among diet based therapies [9]. Dates are good options in this regard due to their wide ranging health promoting parameters and are abundantly produced [10]. Considering the aforementioned essentials this project was designed to produce a nourishing date bar with commercial value which surely would have superior nutritional profile and wholesomeness to that of existing products. A date based food bar supplemented with chick peas as a cheap source of high quality protein containing lysine an essential amino acid and 22 other amino acids including 8 essential amino acids present in date flesh. Their low moisture can complement the high moisture content (60%) of date flesh giving the date bar suitable matrix without addition of water externally and thereby increasing storability and nuts can be prepared and utilized to reduce the risk factors of obesity [11]. Keeping in view the above facts, the study was designed to explore the physico-chemical attributes and nutritional profile of different indigenous and exotic date varieties. Bio-efficacy of indigenous and exotic date varieties using rats as model organisms and to develop functional date bar and its sensory evaluation.

## Materials and methods

The present study was carried out at Institute of Home and Food Sciences Government College University Faisalabad and Food Technology section, Ayub Agricultural Research Institute, Faisalabad, Pakistan. This study comprises of three phases, physiochemical profiling, functional evaluation and finally bio-efficacy study through rats. The material used and procedures adopted are described as under:

### Procurement of raw materials

Four date varieties three indigenous and one exotic were used in the present study. Indigenous varieties includes Hallawi Aseel and Khudravi were collected from Date Palm Research Sub-Station, Jhang-Pakistan and one exotic which is Ajwa was imported from Saudi Arabia. Various analytical and HPLC grade reagents and standards were purchased from Sigma Aldrich (Seelze, Germany), E. Merck (Darmstadt, Germany). Moreover, the male albino rats were purchased from National Institute of Health (NIH) Islamabad. All the raw materials were analyzed according to the following standard methods as described below.

### Chemical assay

Moisture content in samples was determined according to the hot air oven method as described in AOAC [12]. Crude protein percentage in the samples was estimated using the Kjeldahl’s method AOAC [12]. Crude fat was determined by solvent extraction method using soxhlet apparatus as mentioned in AOAC [12]. Crude fiber in the samples was estimated by following the standard procedure as given in AOAC [12]. Total ash was determined by incineration of sample as inorganic matter by following the procedure as mentioned in AOAC [12]. The NFE was calculated by difference using following formula:

$$ \mathrm{NFE}\ \left(\%\right)=100-\left(\%\mathrm{CP}+\%\mathrm{EE}+\%\mathrm{CF}+\%\mathrm{Ash}\right) $$

### Physical analysis of different date varieties

#### Texture and weight analysis

Texture and fruit weight of the different date varieties were determined according to the method given by Piga et al [13].

### Minerals estimation

Sample for mineral analysis was prepared by the wet digestion method. 0.5 g of dried date sample was first digested at low temperature (60-70 °C) with 10 mL HNO_3_ for 20 min in a 100 mL conical flask on hot plate, and then it was digested at high temperature (190 °C) with 5 ml 60% HCLO_4_ till the contents of flask become clear. The digested sample was transferred to 100 mL volumetric flask and volume was made with double distilled and deionized water and then filtered [14]. The filtered sample solution was run by the atomic absorption spectrophotometer. Sample of known strength were first run for each mineral to obtain standard curve. The mineral contents of the samples were determined by using the respective standard curve prepared for each element described in AACC [15]. Sodium and potassium were estimated by flame photometer according to the method given in AOAC [12].

### Antioxidant profile

#### DPPH free radical scavenging assay

2, 2-Diphenyl-1-picrylhydrazyl radical (DPPH•) assay was carried out to measure the free radical scavenging activity of the samples as described by Rababah et al [16]. The extract of date varieties in methanol (1–100 μg/mL) were mixed with 2 mL of 90 μM methanol solution of DPPH. After 30 min incubation period at room temperature, the absorbance was read at 517 nm. Butylated hydroxytoluene (BHT) and butylated hydroxyl anisole (BHA) were used as positive control for comparison and 90 μM DPPH solutions was taken as blank. The percent scavenging was calculated by following formula:

$$ \mathrm{Inhibition}\ \left(\%\right)=\left(\frac{A_{blank-{A}_{sample}}}{A_{blank}}\right)\times 100 $$

Where A_blank_ is the absorbance of the DPPH solution and A_sample_ is the absorbance of the extract solution.

#### Total phenolics contents (TPC)

Date fruit sample (25 g) of each variety was extracted with 100 mL of 80% (v/v) ethanol in a mechanical shaker for 6 h. It was centrifuged at 10,000 rpm for 20 min. The supernatant was filtered using Whatman No. 1 filter paper and evaporated to dryness at 40 °C under vacuum in a rotary evaporator. The dried extract obtained was again diluted to 100 ml with distilled water and stored in an air tight container at -21 °C until further use. Total phenols were analyzed with UV spectrophotometer (Specord®200 Plus Germany) by Folin-Ciocalteu reagent method as described by Singh et al [17]. From prepared sample solution 125 μl of sample were taken in a test tube. 500 μl distilled water and 125 μl of Folin-Ciocalteu reagent were added in it and gave a stand of 6 min. Then 1.25 mL of sodium carbonate (7%) was added in it. Final volume was made to 3 mL by adding 1 mL distilled water. The samples were incubated at room temperature for 120 min for completion of reaction. The absorbance of the samples was measured in triplicate at 760 nm by using a UV-VIS spectrophotometer. Gallic acid was run to prepare standard curve and its absorbance was taken at 725 nm. 0.5 g Gallic acid was dissolved in 10 mL ethanol and then diluted to 100 mL with distilled water (5 g/L). Dilutions of 1, 2, 5 and 10 ml to 100 mL with distilled water were prepared to create standards curve with 50, 100, 250 and 500 mg/L concentrations, respectively and its standard curve was used for the calculation of total phenolic contents in the samples.

### Reducing sugar

Reducing sugars were determined according to Lane and Eynon method as described in method No. 925–36 of AOAC [18]. Reducing sugars have free aldehyde or Ketone group and have the ability to reduce the copper in Fehling solution to brick red insoluble Cuprous Oxide. The sugar content in date fruit sample is estimated by determining the volume of unknown sugar solution required to completely reduce the measured volume of Fehling’s solution.

### Efficacy study

A bio-efficacy study of 56 days was conducted on 6 groups of Sprague Dawley rats, each group having 6 rats, to evaluate the impact of date paste from different varieties in suppressing obesity. 36 Male albino rats of 8–9 weeks were procured and housed in the animal room of Department of Pharmacy, Government College University Faisalabad. Initially, the rats were acclimatized by feeding basal diet for one-week period. During the experiment, the environmental conditions were maintained i.e. temperature 23 ± 2 °C and relative humidity 55 ± 5% with 12 h light-dark period. In the animal modeling, six groups of rats were shaped assigning 6 rats in each group and through simultaneous synchronization obesity was induced in all 5 groups of rats through high cholesterol high sucrose diet. Group 3 to group 6 were fed with high cholesterol high sucrose diet + date paste intervention except group 1 which is control. The experimental rats were monitored for the feed and water intake along with body weight gain throughout the trial. At the termination (56th day) of trial overnight fasted rats were decapitated and blood samples were collected in EDTA coated tubes. Furthermore, the serum was separated after centrifuging the blood through centrifuge machine for 6 min @ 4000 rpm. The collected serum samples were kept for biochemical evaluation through Microlab 300 (Merck, Germany). The results were inferred statistically to establish a conclusive approach.

### Blood assay

Blood assay including total cholesterol, high density lipoproteins, low density lipoproteins and triglycerides, urea, creatinine and Alanine aminotransferase **(**ALT) were measured according to their respective protocols. Total cholesterol levels of the rat’s serum samples were determined using CHOD–PAP method following the protocol of Stockbridge et al [19]. High density lipoprotein (HDL) samples were measured by HDL Cholesterol Precipitant method as mentioned by Assmann [20]. Low density lipoproteins (LDL) were determined by following the procedure of McNamara et al [21]. Total triglycerides in all samples were determined by liquid triglycerides (GPO–PAP) method as outlined by Annoni et al [22]. Blood urea and creatinine (GLDH-method) were determined using commercial kits Jacobs et al [23] to assess the renal functionality. Alanine aminotransferase (ALT) was also assessed. Level of ALT was measured by the dinitrophenyl hydrazine (DNPH) method using Sigma Kits 59–50 and 58–50 described by Basuny [24] to assess liver function test. On the basis of bioefficacy study best date variety having high anti-obesity perspective was used further for the development of date bar.

### Preparation of date bars

#### Pre-treatment of dates

Dates were pitted, washed and dried. Pitted dates were steamed for 3 min. These were then dried. Peanuts and almond were shelled, skin removed and crushed to form grits. Roasted gram was ground to form flour.

#### Development of date bars

After preparation of raw materials, dates were passed through mincing machine to make paste. Other ingredients (roasted gram flour, skim milk, peanuts and almonds) were mixed thoroughly to distribute uniformly and to make a blend. After mixing sheeting was done this was cut into bars of 2.5 cm width and 7 cm in length. The quantity of roasted gram flour, skim milk, peanuts and almonds remained constant while date paste was used in different proportions.

### Sensory evaluation

The sensory evaluation of the four treatments of prepared date bars was conducted by a trained taste panel of 10 judges for attributes like color, flavor, taste, texture, mouth feel and overall acceptability following the procedure of Heintz and Kader [25]. All evaluations were conducted at room temperature under white light. Panelists were provided with distilled water to rinse their mouths between the samples. The samples were presented in random order and panelists were asked to rate their acceptance by giving a score for all the parameters.

### Statistical analysis

The data obtained for different parameters were analyzed statistically by applying Completely Randomized Design (CRD). Levels of significance (*P* ≤ 0.05) were determined by applying ANOVA by following the principles outlined by Steel et al [26]. The means were compared by using LSD.

## Results and discussion

### Proximate composition

Proximate composition is a key factor for assessing the quality of raw material. The results reveal that ash content in four date varieties ranged from 1.68 ± 0.04% to 2.15 ± 0.19%. Higher ash content was observed in Khudravi (2.15 ± 0.19%) trailed by Hallawi (1.90 ± 0.03%) and minimum was observed in both Aseel and Ajwa. Crude protein content in four different date varieties ranged from 2.49 ± 0.13% to 2.77 ± 0.04%. Maximum values were recorded in Ajwa (2.77 ± 0.04%) followed by Aseel (2.77 ± 0.04%) and minimum was found in Hallawi (2.49 ± 0.13%) (Table 1). Crude fat content ranged from 0.26 ± 0.03% to 0.55 ± 0.03%. Maximum crude fat content was observed in Khudravi (0.55 ± 0.03%) followed by Hallawi (0.49 ± 0.04%) and minimum was observed in Ajwa (0.26 ± 0.03%). Crude fibre contents ranged from 2.02 ± 0.13% to 2.64 ± 0.07% and higher values were recorded in Aseel (2.64 ± 0.07%) trailed by Khudravi (2.47 ± 0.05%) and minimum was observed in both Ajwa and Hallawi (2.02 ± 0.13%). Due to similar processing conditions the moisture contents were observed to be more or less similar and ranged from 22.99 ± 0.02% to 25.75 ± 0.03%. Higher moisture content was observed in Aseel and Ajwa (25.75 ± 0.03%) followed by Khudravi (24.85 ± 0.06%) and Hallawi (22.99 ± 0.02%). Likewise, the recorded NFE values were ranged from 92.18 ± 0.13% to 93.08 ± 0.20 g %. Higher NFE was recorded in Hallawi (93.08 ± 0.20%) trailed by Ajwa (93.08 ± 0.16%) and minimum was observed in Khudravi (92.18 ± 0.13%) variety as shown in Table 1. The results of the present study are in agreement with findings of Nadeem et al [27] who narrated the values for moisture, crude protein, crude fat, crude fiber, ash and NFE as 24.79 g/100 g, 2.55 g/100 g, 0.47 g/100 g, 3.62 g/100 g, 1.31 g/100 g and 92.06 g/100 g respectively for different date varieties. Likewise, Al-Shahib and Marshal [11] had also carried out proximate profiling and observed that percent values of moisture content of different dates ranged between 9.2 to 32.1%, crude protein in the range of 1.7 to 3.0%, crude fat ranged from 0.1 to 0.5%, crude fiber in the range from 1.7 to 4.6%, and ash in the range of 0.3 to 2.4%. The results of this study are also in correspondence with Ismail et al [28] who evaluated the date varieties for their percent moisture, crude protein and ash as 20.25 to 22.14%, 2.3 to 2.7% and 1.83 to 2.36%.

**Table 1:** Proximate composition of different date varieties

Varieties	Ash %	Moisture %	Fat %	Fiber %	Protein %	NFE %
Ajwa	1.68 ± 0.05^c^	25.75 ± 0.04^a^	0.26 ± 0.01^b^	2.43 ± 0.04^a^	2.77 ± 0.01^a^	93.08 ± 0.02^a^
Aseel	1.68 ± 0.04^c^	25.75 ± 0.03^a^	0.28 ± 0.05^b^	2.64 ± 0.04^a^	2.77 ± 0.04^a^	92.62 ± 0.20^b^
Hallawi	1.90 ± 0.03^b^	22.99 ± 0.02^c^	0.49 ± 0.04^a^	2.02 ± 0.03^b^	2.49 ± 0.13^b^	93.08 ± 0.20^a^
Khudravi	2.15 ± 0.19^a^	24.85 ± 0.06^b^	0.55 ± 0.03^a^	2.47 ± 0.02^a^	2.64 ± 0.02^a^	92.18 ± 0.13^c^

Different letters showed significant variations (*p* ≤ 0.05) in column; values are expressed as means ± standard deviation of three independent determinations

Similarly, Yosef and Kado [29] evaluated Hallawi and Khudravi for their moisture content as Hallawi (7.30 g/100 g) followed by Khudravi (9.50 g/100 g), protein value for Hallawi (2.30 g/110 g) trailed by Khudravi (2.43 g/110 g), fat content in Hallawi (0.51 g/100 g) and Khudravi (0.47 g/100 g), ash content in Hallawi (1.92 g/100 g), Khudravi (2.12 g/100 g) and crude fiber in Hallawi (1.82 g/100 g) followed by Khudravi (2.28 g/100 g) and these results are in compliance with the present study. The slight variations in proximate composition of instant findings might be due to various factors, basically date fruit matures after passing through four main ripening stages kimri (unripe), khalal (full-size, slightly crunchy; edible), rutab (ripe, soft; edible), and tamar (ripe, reduced moisture; edible) [30]. Many researchers are of view that as date fruit progresses towards maturity ash, fibre and moisture content follow a decreasing trend. This reduction is due to the common dates preservation method of sun drying. Moisture content also depends upon the stage of ripening and it decreases as it ripened for further stages from Kimri to Tamar [31].

### Texture analysis

Results regarding mean values of texture analysis and fruit weight were showed in Table 2. Mean value for texture of four different date varieties ranged from 30.13 ± 0.01 g to 38.39 ± 0.03 g. The highest value of texture was found in Ajwa (38.39 ± 0.03 g), followed by Aseel (37.40 ± 0.02 g) and minimum was observed in Khudravi in Table 2. Similarly fruit weight of different date varieties ranged from 6.89 ± 0.01 g to 11.68 ± 0.01 g. Results for Fruit weight of four date verities showed maximum values for Hallawi **(**11.68 ± 0.01 g), followed by Khudravi (11.58 ± 0.01 g) and minimum was observed in Aseel (6.89 ± 0.01 g) as shown in Table 2. The present outcomes for texture and weight are in close agreement with the findings of Nadeem et al [32] who investigated that twenty-one date palm varieties for fruit weight among them Aseel and Hillawi were (6.98 g) and (11.72 g), respectively and the maximum value of texture was recorded in Aseel (38.49 g) followed by Hallawi (35.32 g). In another study conducted by Khan et al [33] where they observed the physical characteristics of different date varieties. Highest fruit weight was found in Dhakki (13.89 g) followed by Dora Basraywal (11.87 g) and Hillawi (11.72 g) and minimum weight was observed in Desi simple (3.04 g).

**Table 2:** Texture analysis and fruit weight of different date varieties

Varieties	Texture (g)	Fruit Weight (g)
Ajwa	38.39 ± 0.03^a^	11.41 ± 0.01^c^
Hallawi	31.58 ± 0.03^c^	11.68 ± 0.01^a^
Aseel	37.40 ± 0.02^b^	6.89 ± 0.01^d^
Khudravi	30.13 ± 0.01^d^	11.58 ± 0.01^b^

Different letters showed significant variations (*p* ≤ 0.05) in columns; values are expressed as mean ± standard deviation of three independent determinations

### Mineral profile

During the present study, different macro and micro minerals were determined in different date varieties. The mineral analysis of 4 different date varieties elucidated that calcium, iron, magnesium, potassium and zinc were present in momentous amounts whereas, non-momentous trend was observed among sodium. The highest value was found in Ajwa (191.00 ± 1.07 mg/100 g), followed by Aseel (160.03 ± 1.02 mg/100 g) and minimum was observed in Khudravi (135.03 ± 0.95 mg/100 g) as shown in Table 3. Similarly, higher sodium content was observed in Aseel (18.00 ± 1.06 mg/100 g) followed by Hallawi (12.00 ± 1.04 mg/100 g) and minimum was found in Ajwa (9.50 ± 0.1 mg/100 g). The iron content found to be higher in Hallawi (5.40 ± 0.01 mg/100 g) followed by Khudravi (4.40 ± 0.01 mg/100 g) and minimum was found in Ajwa (3.13 ± 0.01 mg/100 g). Magnesium content found to be high in Ajwa (146.93 ± 0.01 mg/100 g) followed by Aseel (56.00 ± 0.02 mg/100 g) and minimum was observed in Hallawi (50.32 ± 0.02 mg/100 g) as presented in Table 3. Maximum value for potassium was found in Khudravi (887.20 ± 1.02 mg/100 g) followed by Hallawi (849.33 ± 0.98 mg/100 g) and minimum was observed in Aseel (465.00 ± 2.95 mg/100 g). The amount of zinc was higher value was noticed in Aseel (1.80 ± 0.01 mg/100 g) followed by Hallawi (1.40 ± 0.01 mg/100 g) and minimum was observed in Khudravi (1.27 ± 0 mg/100 g) in Table 3. The results of present findings are in harmony with the results documented earlier by Assirey [34] who reported that different varieties of date fruit contain highest amount of potassium ranged from 289.6–512 mg/100 g, calcium 123-187 mg/100 g, magnesium ranged between 56 and 150 mg/100 g and sodium ranged from 4.9–8.9 mg/100 g. Nasir et al [31] has evaluated that date fruit contain potassium as (713 mg/100 g), magnesium as (64.2 mg/100 g), copper as (0.24 mg/100 g) and selenium as (0.31 mg/100 g) respectively. The present outcomes are also in accordance with Yousef and Kado [30] who reported mineral composition for different date varieties including hallawi and Khudravi for their macro and micro minerals. Hallawi contains (184 mg/100 g) calcium, (854 mg/100 g) potassium, (14 mg/100 g) sodium, (56 mg/100 g) magnesium, (5.26 mg/100 g) iron and (1.39 mg/100 g) zinc. Khudravi contains (133 mg/100 g) calcium, (894 mg/100 g) potassium, (16 mg/100 g) sodium, (60 mg/100 g) magnesium, (4.5 mg/100 g) iron, (1.29 mg/100 g) zinc. Vinita and Punia [35] depicted that the results are in line with the current findings. Maximum values of potassium (853.33 mg/100 g), calcium (159.66 mg/100 g), magnesium (53.33 mg/100 g), iron (5.34 mg/100 g) and zinc (1.41 mg/100 g) for Hallawi were observed. Similarly, for Khudravi highest value of potassium (893.66 mg/100 g), calcium (139.00 mg/100 g), magnesium (58.33 mg/100 g), iron (4.45 mg/100 g) and zinc as (1.2 5 mg/100 g). Assirey [34] narrated the mineral composition of Ajwa date fruit as potassium (476.3 mg/100 g), calcium (187 mg/100 g), magnesium (150 mg/100 g), sodium (7.5 mg/100 g) and these results are in line with the present outcomes for Ajwa date fruit. The variations in outcomes observed in the present studies might be related to climate, soil type, varietal differences and developmental stage. The transition of date fruit from Kimri to the tamar stage is associated with a reduction in the amounts of all minerals [30, 36–38]. The percentages of calcium, magnesium, phosphorus, potassium, sodium and zinc decreased from Kimri to Tamar stage.

**Table 3:** Mineral composition of different date varieties (mg/100 g)

Varieties	Sodium	Calcium	Iron	Magnesium	Potassium	Zinc
Ajwa	9.50 ± 0.1^d^	191.00 ± 1.07a	3.13 ± 0.01^d^	146.93 ± 0.01^c^	482.00 ± 2.98^c^	1.30 ± 0.01^b^
Aseel	18.00 ± 1.06^a^	160.03 ± 1.02^b^	3.30 ± 0.01^c^	56.00 ± 0.02^ab^	465.00 ± 2.95^d^	1.80 ± 2.01^a^
Hallawi	12.00 ± 1.04^b^	165.66 ± 0.99^b^	5.40 ± 0.01^a^	50.32 ± 0.02^b^	849.33 ± 0.98^b^	1.40 ± 0.01^b^
Khudravi	11.00 ± 1.02^c^	135.03 ± 0.95^c^	4.40 ± 0.01^a^	52.35 ± 0.01^a^	887.20 ± 1.02^a^	1.27 ± 0.01^b^

Different letters showed significant variations (p ≤ 0.05) in columns; values are expressed as mean ± standard deviation of three independent determinations

### Antioxidant profile

Polyphenols are the most abundant antioxidants in our diet and are extensive constituents of fruits, cereals, vegetables, legumes, coffee and tea. As antioxidants, polyphenols may protect cell constituents against oxidative damage and, therefore, limit the risk of various degenerative diseases associated to oxidative stress [39]. In plants, phenolic and polyphenolic compounds represent the main class of natural antioxidants that are directly responsible for anti-oxidative action [40]. The highest value of TPC was found in Aseel (291.36 ± 0.04 mg/100 g) trailed by Hallawi (282.65 ± 0.04 mg/100 g) and lowest was found in Khudravi (232.64 ± 0.07 mg/100 g) Table 4. It has been proved through studies that many fruits and vegetables exhibit total antioxidant activity significantly owing to the presence of many flavonoids and related polyphenols [41]. Therefore, these four varieties of dates (Hallawi, Ajwa, Khudravi and Aseel) were probed for its free radical scavenging activity. Maximum value of free radical scavenging activity was observed in Aseel (89.15 ± 1.65%) followed by Khudravi (84.65 ± 1.65%) and minimum was observed in Hallawi (66.14 ± 2.55%) as shown in Table 4. The outcomes of present study for phenolic compounds in different date varieties are in line with Al-Farsi and Lee et al [42] who narrated phenolics of dates ranged from 193.7 mg/100 g for fresh dates to 239.5 mg/100 g for dried dates. Saleh et al [43] reported the concentration of total polyphenols in three Saudi premium quality date varieties (Ajwa, Sukari, Khalal). According to him polyphenols concentration depends upon date variety and extraction solvent. Polyphenols in the aqueous extract were significantly higher compared to the alcohol extract. Ajwa water extract contains polyphenols as (455.88 mg/100 g) and Ajwa alcohol extract contain polyphenols as (245.66 mg/100 g). Sukari water extract contains polyphenols as (377.66 mg/100 g) and Sukari alcohol extract contain polyphenols as (222.7 mg/100 g). Khalal water extract contains polyphenols as (238.54 mg/100 g) and Khalal alcohol extract contain polyphenols as (106.06 mg/100 g). Total phenolic content of Ajwa fruit varied between 245 and 455 mg/100 g. Ajwa date contain higher phenolic contents at Kimri stage (290 mg/100 g) followed by Khalal (150 mg/100 g), Rutab (20 mg/100 g) and at Tamar stage (10 mg/100 g). Mansouri et al [44] and Biglari et al [45] reported that total phenolic content ranged from 2.49 to 8.36 mg/100 g Gallic acid equivalents per 100 g of fresh weight of Algerian and Iranian dates, respectively. Saafi et al [46] recorded the phenolic contents of dates derived from four commercial varieties. The analysis showed that the total phenolic in Kentichi on fresh weight basis ranged from 209.4 mg/100 g of Gallic acid equivalents and 447.7 mg/100 g of Gallic acid equivalent. Present findings are also in correspondence with Nadeem et al [32] who narrated the total phenolic values of some Pakistani date varieties on fresh basis as in Dhakki (296.67 mg/100 g) followed by Aseel (283.33 mg/100 g) Hallawi (275.67 mg/100 g). The lowest mean value of total phenols was observed in Desi red small (140.67 mg/100 g) followed by Desi black (148.0 mg/100 g) and Shungust (163.33 mg/100 g). Antioxidant activity of present study was in compliance with Anjum et al [47] who has observed three Pakistani date varieties for their antioxidant activity by DPPH in methanol extract. Highest antioxidant activity was found in Karbalane as (90.965%) followed by Dora (89.79%) and Dhaki as (85.75%).

**Table 4:** Antioxidant profile of different varieties

Varieties	TPC (mg/100 g)	% DPPH scavenging
Ajwa	252.65 ± 0.05^c^	75.13 ± 2.78^c^
Hillawi	282.65 ± 0.04^b^	66.14 ± 2.55^d^
Aseel	291.36 ± 0.04^a^	89.15 ± 1.65^a^
Khudravi	232.64 ± 0.07^d^	84.65 ± 1.65^b^

Different letters show significant variations (*p* ≤ 0.05) in columns; values are expressed as means ± standard deviation in three independent determinations

### Sugar profile

Maximum value for total sugar content was observed in Ajwa (86.24 ± 1.54%) followed by Hallawi (83.58 ± 2.41%) and minimum was recorded in Aseel (71.96 ± 1.42%) as shown in Table 5. Reducing sugar content in four different date varieties ranged from 68.18 ± 1.39% to 79.45 ± 1.22%. Highest mean value for reducing sugar content were observed in Ajwa (79.45 ± 1.22%) followed by Hallawi (77.68 ± 1.42%) and minimum was found in Aseel (68.18 ± 1.39%). Non-reducing sugars ranged between 3.78 ± 0.02% to 7.13 ± 0.02%. Higher content of non-reducing sugars was recorded in Khudravi (7.13 ± 0.02%), followed by Ajwa (6.74 ± 0.01%) and minimum was recorded in Aseel (3.78 ± 0.02%) as presented in Table 5. Results regarding total sugars, reducing sugars and non-reducing sugars in the present study corroborate with the previous findings of Nadeem et al [27] who narrated the values of total sugars, reducing sugars and non-reducing sugars of different Pakistani date varieties. Maximum value for total sugars ranged between 59.03–73.92%. Karblain (73.92%), Zaidy (72.87%), Aseel (67.11%) and Hallawi (67.16%). Reducing sugars ranged from 52.76–68.95%. Karblain (68.95%), Zaidy (967.13%), Aseel (62.46%), Hallawi (59.50%). In case of non-reducing ranged vary between 4.65–7.66%. Karblain (4.97%), Zaidy (5.72%), Aseel (4.65%), Hallawi (7.66%) respectively. Ismail et al [29] has studied two different date varieties and observed that reducing sugars ranged between 69.9–75.2%. Current findings are strongly in line with Ramadan [48] reported that reducing sugars in different date varieties ranged from 70.28–80.52%, non-reducing ranged from 0.59–3.28% and total sugars ranged between 70.87–83.80% respectively. Assirey [34] had reported the sugar composition of 10 date fruit cultivars in which Ajwa contains total sugars (74.3%) and reducing sugars (71.1%). Yousef and Kado [30] reported Hallawi and Khudravi for their total and reducing sugars. Hallawi (87.91%) total sugars and (82.72%) reducing sugars followed by Khudravi contains (87.74%) total sugars and (81.91%) reducing sugars. Present outcomes of this study are in correspondence with Vinita and Punia [35] who observed Hallawi and Khudravi for their total, reducing and non-reducing sugars. In Hallawi total sugars (82.50%), reducing sugars (76.60%) and non-reducing sugars (5.82%) trailed by Khudravi, total sugars (81.39%), reducing sugars (75.24%) and non-reducing sugars (6.15%).

**Table 5:** Total, reducing and non-reducing sugars of different date varieties

Varieties	Total sugars %	Reducing sugar %	Non reducing % sugars
Ajwa	86.24 ± 1.54^a^	79.45 ± 1.22^a^	6.74 ± 0.01^b^
Hallawi	83.58 ± 2.41^a^	77.68 ± 1.42^a^	5.90 ± 0.01^b^
Aseel	71.96 ± 1.42^c^	68.18 ± 1.39^c^	3.78 ± 0.02^c^
Khudravi	80.46 ± 2.63^b^	73.33 ± 1.32^b^	7.13 ± 0.02^a^

Different letters showed significant variations (*p* ≤ 0.05) in columns; values are expressed as means ± standard deviation in three independent determinations

Sugar level of date fruits increases as dates move from Rutab to Tamar stage because of dryness. Dried and fresh dates levels of sugars variations due to stages of maturity and moisture reduction. Glucose (reducing sugars) results in fast elevation of blood glucose level than sucrose. Fructose sugar two times sweeter than glucose exert feeling of fullness and reduce total caloric intake than consuming fat rich diets is more suitable for obese patients [34].

### Bio-efficacy study

Four date varieties, three indigenous (Aseel, Hallawi and Khudravi) and one exotic (Ajwa) were subjected to efficacy trials through male albino rats modeling to assess its nutraceutical potential against lifestyle-related disorders. Ease in handling, organized supervision, controlled diet and environmental conditions were the key factors to conduct experimental trials in rodents rather than humans. Efficacy study was divided into six modules on the basis of different diets i.e. group I (normal diet), group II (high cholesterol high sucrose diet), group III (high cholesterol high sucrose diet+ Khudravi date variety), group IV (high cholesterol high sucrose diet + Hallawi date variety), group V (high cholesterol high sucrose diet + Aseel date variety) and group VI (high cholesterol high sucrose diet + Ajwa date variety). In each group the respective diet was given to the rat groups along with date paste intervention in a proportion as selected from sensory and compositional study. Role of dates against obesity, high cholesterol, high triglyceride, HDL, LDL and oxidative stress was observed. The results of the investigated parameters in all studies were interpreted collectively for better understanding of concern.

#### Total cholesterol

Higher value (148.33 ± 0.57 mg/dL) of total cholesterol was found in T_1_ (high cholesterol high sucrose diet) trailed by _(_136.67 ± 1.54 mg/dL) in T_2_ (high cholesterol high sucrose diet + Khudravi date variety) and minimum (83.66 ± 0.57 mg/dL) was found in T_0_ (control) as presented in Table 6. In T_0_ the value was lowest as they are not fed on any experimental diet. T_1_ has the highest value of cholesterol as they were fed on high cholesterol high sucrose diet to induce obesity. In all other groups obesity was induced with intervention of dates of different varieties to investigate the highest cholesterol lowering effect of each date variety. Lowest value (103.67 ± 1.15 mg/dL) of total cholesterol was observed in T_5_ (high cholesterol high sucrose diet + Ajwa date variety). This significant variation in all treatments shows that all varieties have lowers the cholesterol significantly when compared to T_1_ (high cholesterol high sucrose diet). The present outcomes are in harmony with Prasanna et al [49] who narrated that aqueous extract of *Phoenix dactylifera* dates significantly lowers the serum cholesterol level in male wistar rat’s groups (control, high fat diet, high fat diet *Phoenix dactylifera*) extract as 83.0 mg/dL, 156.1 mg/dL, 84.3 mg/dL respectively. In another study Pushpa and Jayachitra [50] has observed the effect of *Phoenix dactylifera* on cholesterol in four groups of Albino wistar rats (control, control+ *Phoenix dactylifera,* Triton, Triton+ *Phoenix dactylifera*) as 82.11 mg/dL, 87.31 mg/dL, 204.51 mg/dL and 92.41 mg/dL respectively. Al-Saif et al [51] investigated that the cholesterol reduction by date palm phytochemicals fed male hamsters by incorporating date pulp in diet at the rate of 50% and observed 11.03% reduction in blood cholesterol when compared with the control group. From the outcomes of present study regarding serum cholesterol level, it is concluded that Polyphenols in date palm fruit are effective for the management of elevated cholesterol level due to consumption of high energy foods.

**Table 6:** Total Cholesterol, Triglycerides, LDL, HDL, ALT, Creatinine and Urea in animal blood

Parameters	Treatments					
	T_0_	T_1_	T_2_	T_3_	T_4_	T_5_
Cholesterol	83.66 ± 0.57^f^	148.33 ± 0.57^a^	136.67 ± 1.54^b^	110.67 ± 1.15^d^	130.33 ± 0.57^c^	103.67 ± 1.15 ^e^
Triglyceride	69.39 ± 0.44^f^	110.67 ± 1.15^a^	92.33 ± 0.57^c^	76.83 ± 1.04^d^	100.67 ± 0.57^b^	72.33 ± 0.57^e^
LDL	27.33 ± 0.57^f^	92.86 ± 0.98^a^	70.66 ± 1.15^b^	55.33 ± 0.57^e^	66.66 ± 1.15^c^	60.00 ± 1.73^d^
HDL	56.66 ± 0.57^d^	39.86 ± 1.15^a^	52.66 ± 1.15^e^	62.33 ± 0.57^c^	57.66 ± 1.15^d^	84.66 ± 1.15^b^
ALT	42.66 ± 0.57^f^	58.66 ± 1.15^a^	53.66 ± 1.15^b^	48.66 ± 0.57^d^	50.66 ± 1.15^c^	45.33 ± 1.15^e^
Creatinine	0.78 ± 0.01^c^	1.72 ± 0.01^a^	1.30 ± 0.17^b^	0.83 ± 0.05^c^	0.43 ± 0.11^d^	0.76 ± 0.11^c^
Urea	21.96 ± 0.35^e^	37.66 ± 1.15^a^	33.66 ± 1.15^b^	27.66 ± 1.15^d^	30.66 ± 0.57^c^	27.33 ± 0.57^d^

Different letters show significant variations (*p* ≤ 0.05) in rows; values are expressed as mean ± standard deviation of three independent determinations

T_0_: Control

T_1_: High sucrose high cholesterol diet

T_2_: High sucrose high cholesterol diet + Intervention (Khudravi)

T_3_: High sucrose high cholesterol diet + Intervention (Hallawi)

T_4_: High sucrose high cholesterol diet + Intervention (Aseel)

T_5_: High sucrose high cholesterol diet + Intervention (Ajwa)

#### Low density lipoprotein

Higher value (92.86 ± 0.98 mg/dL) of LDL was found in T_1_ (high cholesterol high sucrose diet) trailed by T_2_ (high cholesterol high sucrose diet + Khudravi date variety) (70.66 ± 1.15 mg/dL) and minimum (27.33 ± 0.57 mg/dL) was found in T_0_ (control) as presented in Table 6. In T_0_ the value was lowest as they are not fed on any experimental diet. T_1_ has the highest value of LDL as they were fed on high cholesterol high sucrose diet to induce obesity. In all other groups obesity was induced with intervention of dates of different varieties to investigate the highest LDL lowering effect of each date variety. Lowest value (55.33 ± 0.57 mg/dL) of LDL was observed in T_3_ (high cholesterol high sucrose diet + Hallawi date variety). This significant variation in all treatments shows that all varieties have lowers the LDL when compared to T_1_ group (high cholesterol high sucrose diet). Research conducted by Bursill et al [52] who observed that at 50% diet supplementation with date pulp 37.27 mg/dL reduction in LDL level. Low density lipoproteins lowering potential of date fruit is attributed due to the presence of bioactive polyphenols most important phenolics and flavonoids. Function of polyphenols is to up regulate LDL receptor to lower blood cholesterol level. As much as these receptors will increase more LDL cholesterol will be up taken from blood circulation [52]. It is evident from the above discussion that Ajwa and Hallawi has more potential to be introduced as dietary intervention against elevated LDL and other lipid related abnormalities. The findings of present research are in accordance with Prasanna et al [50] who narrated that aqueous extract of *Phoenix dactylifera* dates significantly lowers the serum LDL level in male Wistar rat’s groups (control, high fat diet, high fat diet *Phoenix dactylifera*) extract as 27.5 mg/dL, 94.3 mg/dL, 31.5 mg/dL respectively. Pushpa and Jayachitra [51] has recorded the effect of *Phoenix dactylifera* on LDL in four groups of Albino Wistar rats (control, control+ *Phoenix dactylifera,* Triton, Triton+ *Phoenix dactylifera*) as 26.18 mg/dL, 30.70 mg/dL, 158.59 mg/dL and 31.93 mg/dL respectively.

#### High density lipoproteins

Higher value (84.66 ± 1.15 mg/dL) of HDL was found in T_5_ (high cholesterol high sucrose diet + Ajwa date variety) trailed by (62.33 ± 0.57 mg/dL) in T_3_ (high cholesterol high sucrose diet + Hallawi date variety) and minimum (39.86 ± 1.15 mg/dL) was found in T_1_ (High cholesterol high sucrose diet) to induce obesity which significantly lowers the HDL level, as presented in Table 6. In all other groups obesity was induced with intervention of dates of different varieties to investigate the highest HDL increasing effect of each date variety. Highest value (84.66 ± 1.15 mg/dL) of HDL was observed in T_5_ (high cholesterol high sucrose diet + Ajwa date variety). This significant variation in all treatments showed that all varieties increased the HDL. Cholesterol in blood is transported via small packages called lipoproteins i.e. LDL and HDL. HDL is considered as good cholesterol as they carry toxins and free fatty acids from blood stream to liver where they are eliminated from the system thus reduces the chances of increase in plasma cholesterol and prevents the occurrence of chronic diseases [53]. LDL on the other hand facilitates cholesterol circulation in blood stream [54]. The outcomes of current study are in accordance with the findings of Al-Saif et al [52] who reported that after administering 50% supplemented rat diet with date pulp increases 8.63% HDL level. All above discussed varieties significantly increases HDL level more prominently Ajwa and Hallawi thus advocating their use as functional food or functional food ingredient. The findings of present research are in accordance with Prasanna et al [50] who narrated that aqueous extract of *Phoenix dactylifera* dates significantly increase the HDL level in male Wistar rat’s groups (control, high fat diet, high fat diet *Phoenix dactylifera*) extract as 58.3 mg/dl, 39.6 mg/dl, 56.4 mg/dl respectively. Pushpa and Jayachitra [51] has recorded the effect of *Phoenix dactylifera* on HDL in four groups of Albino Wistar rats (control, control+ *Phoenix dactylifera,* Triton, Triton+ *Phoenix dactylifera*) as 24.70 mg/dl, 25.61 mg/dl, 19.08 mg/dl and 24.78 mg/dl respectively.

#### Triglycerides

Higher value (110.67 ± 1.15 mg/dL) of total triglyceride was found in T_1_ (high cholesterol high sucrose diet) trailed by (100.67 ± 0.57 mg/dL) in T_4_ (high cholesterol high sucrose diet + Aseel date variety) and minimum (69.390 ± 0.44 mg/dL) was found in T_0_ (control) as presented in Table 6. In T_0_ the value was lowest as they are not fed on any experimental diet. T_1_ has the highest value of total triglyceride as they were fed on high cholesterol high sucrose diet to induce obesity. In all other groups obesity was induced with intervention of dates of different varieties to investigate the highest total triglyceride lowering effect of each date variety. Lowest value (72.33 ± 0.57 mg/dL) of total triglyceride was observed in T_5_ (high cholesterol high sucrose diet + Ajwa date variety). This significant variation in all treatments shows that all varieties have lowers the total triglyceride when compared to T_1_ group (high cholesterol high sucrose diet).

Prasanna et al [50] narrated that aqueous extract of *Phoenix dactylifera* dates significantly lowers the triglyceride level in male Wistar rat’s groups (control, high fat diet, and high fat diet + *Phoenix dactylifera*) extract as 18.5 mg, 22.06 mg, and 18.7 mg respectively. Pushpa and Jayachitra [51] has observed the effect of *Phoenix dactylifera* on triglyceride in four groups of Albino Wistar rats (control, control+ *Phoenix dactylifera,* Triton, Triton+ *Phoenix dactylifera*) as 55.50 mg, 60.07 mg, 479.40 mg and 71.40 mg respectively. Gastrointestinal lipid digestion and absorption requires the enzymatic hydrolysis of triglycerides, thus, many researchers suggest that, the inhibition of pancreatic lipase is a possible strategy to prevent hyperlipidaemia by decreasing lipid absorption. Polyphenols have been reported to decrease pancreatic lipase activity [55].

#### Urea

Higher value (37.66 ± 1.15 mg/dL) of urea was found in T_1_ (high cholesterol high sucrose diet) trailed by (33.66 ± 1.15 mg/dL) in T_2_ (high cholesterol high sucrose diet + Khudravi date variety) and minimum (21.96 ± 0.35 mg/dL) was found in T_0_ (control) as presented in Table 6. In T_0_ the value was lowest as they are not fed on any experimental diet. T_1_ has the highest value of urea as they were fed on high cholesterol high sucrose diet to induce obesity. In all other groups obesity was induced with intervention of dates of different varieties to investigate the highest urea lowering effect of each date variety. Lowest value (27.33 ± 0.57 mg/dL) of urea was observed in T_5_ (high cholesterol high sucrose diet + Ajwa date variety). This significant variation in all treatments shows that all varieties have lowers the urea level significantly when compared to T_1_.

Agarwal et al [56] has narrated that in human body kidney is a major organ which involved in several life sustaining functions including Blood pressure regulation, body homeostatic, and toxins removal in the form of urine from body. In the recent era rapid increase of chronic diseases and chronic renal disease in developing countries is due to bad dietary habits, chemical and toxins exposure. Amira et al [57] has narrated that in male Wistar rats 0.5 and 2 g/L DCA was administered as drinking water to induce nephrotoxicity. Some of the experimental rats were gavage aqueous date extract (ADE) before administrating DCA. When after two months of experiment results were evaluated as those rats fed with DCA caused increased level of renal MDA with considerable depletion of GSH levels and significantly altered the antioxidant enzymes activities. By elevating the levels of plasma urea, uric acid and creatinine levels it affects the renal functioning as compared to control rats. On the other side the ADE treatment normalized the increased plasma levels of creatinine, urea and uric acid, reduced the elevated MDA levels, significantly normalized the antioxidant enzyme activities and GSH level and restored the altered kidney histology in rats treated significantly. Nephrons lost their structural integrity and leads towards reduce glomerulus filtration and increase in urea and creatinine level. This risk is more prevalent in patients suffering from high blood pressure, obese, diabetic and CVDs [58].

#### Creatinine

Higher value (1.72 ± 0.01 mg/dL) of creatinine was found in T_1_ (high cholesterol high sucrose diet) trailed by (1.30 ± 0.17 mg/dL) in T_2_ (high cholesterol high sucrose diet + Khudravi date variety) and minimum (0.43 ± 0.11 mg/dL) was found in T_4_ (high cholesterol high sucrose diet + Aseel date variety) as presented in Table 6. T_1_ has the highest value of creatinine as they were fed on high cholesterol high sucrose diet to induce obesity. In all other groups obesity was induced with intervention of dates of different varieties to investigate the highest creatinine lowering effect of each date variety. Lowest value (0.43 ± 0.11 mg/dL) of creatinine was observed in T_4_ (high cholesterol high sucrose diet + Aseel date variety). This significant variation in all treatments shows that all varieties have lowers the creatinine level significantly when compared to T_1_.

Amira et al [57] has narrated that in male wistar rats 0.5 and 2 g/L DCA was administered as drinking water to induce nephrotoxicity. Some of the experimental rats were gavage aqueous date extract (ADE) before administrating DCA. When after two months of experiment results were evaluated as those rats fed with DCA caused increased level of renal MDA with considerable depletion of GSH levels and significantly altered the antioxidant enzymes activities. By elevating the levels of plasma urea, uric acid and creatinine levels it affects the renal functioning as compared to control rats. On the other side the ADE treatment normalized the increased plasma levels of creatinine, urea and uric acid, reduced the elevated MDA levels, significantly normalized the antioxidant enzyme activities and GSH level and restored the altered kidney histology in rats treated significantly. Nephrons lost their structural integrity and leads towards reduce glomerulus filtration and increase in urea and creatinine level. This risk is more prevalent in patients suffering from high blood pressure, obese, diabetic and CVDs [58].

#### Alanine aminotransferase (ALT)

Higher value (58.66 ± 1.15 U/L) of ALT was found in T_1_ (high cholesterol high sucrose diet) trailed by (53.66 ± 1.15 U/L) in T_2_ (high cholesterol high sucrose diet + Khudravi date variety) and minimum (42.66 ± 0.57 U/L) was found in T_0_ (control) as presented in Table 6. T_1_ has the highest value of ALT as they were fed on high cholesterol high sucrose diet to induce obesity. And T_0_ has the minimum value as they were not fed on any experimental or intervention diet. In all other groups obesity was induced with intervention of dates of different varieties to investigate the highest ALT lowering effect of each date variety. Lowest value (45.33 ± 1.15 U/L) of ALT was observed in T_5_ (high cholesterol high sucrose diet + Ajwa date variety). This significant variation in all treatments shows that all varieties have lowers the ALT level significantly when compared to T_1_. Liver is responsible for the detoxification of human body and act as first line of defense to prevent the deposition on exogenous and endogenous substances. The indicators of liver health are ALT and AST and their elevated level in blood indicates liver malfunctioning. Elevated cholesterol cause increase intracellular lipids deposition and increases the amount of ALT and AST which results in liver functioning failure. Increased level of ROS due to consumption of cholesterol rich diet causes oxidative damage to cells and tissues and structural integrity of the cells damages allowing release of ALT, ALP, AST and bilirubin in blood circulation as they present in cytoplasm [59–61].

### Sensory evaluation

Hedonic response is crucial to judge the product for acceptance and marketability. Good sensory response ensures consumer acceptance and confidence on the developed product. The prepared date bars were assessed for various sensory attributes including color, flavor, taste, mouth feel, texture and overall acceptability and the results gathered are presented herein [25]. Four treatments of date bar from Hallawi were prepared by varying the amount of date paste and chick pea flour while keeping the other ingredients same like skim milk and almonds. Mean values of all the parameters color, flavor, taste, texture, mouthfeel and overall acceptability are shown in Table 7. Scores of color in all treatments ranged from 5.60 ± 0.54 to 8.40 ± 0.54. Highest scores (8.40 ± 0.54) for color given by the panel of judges was recorded in T_2_ (40% Date paste, 30% Chick pea flour) followed by T_3_ (30% Date paste, 40% Chick pea flour) (7.60 ± 0.54) and minimum (5.60 ± 0.54) was recorded in T_4_ (20% Date paste, 50% Chick pea flour) as shown in Table 7. Flavor scores in all four treatments ranged from 5.20 ± 0.44 to 8.40 ± 0.54. Highest scores (8.40 ± 0.54) for flavor given by the panel of judges was recorded in T_2_ (40% Date paste, 30% Chick pea flour) followed by T_3_ (30% Date paste, 40% Chick pea flour) (7.40 ± 0.54) and minimum (5.20 ± 0.44) was recorded in T_4_ (20% Date paste, 50% Chick pea flour) as presented in Table 7. Taste values were ranged from 4.40 ± 0.54 to 8.80 ± 0.44, Highest scores (8.80 ± 0.44) for taste given by the panel of judges was recorded in T_2_ (40% Date paste, 30% Chick pea flour) followed by T_3_ (30% Date paste, 40% Chick pea flour) (7.60 ± 0.54) and minimum (4.40 ± 0.54) was recorded in T_1_ (50% Date paste, 20% Chick pea flour) as presented in Table 7. Texture parameter was ranged between 4.80 ± 0.44 to 8.40 ± 0.54. Highest scores (8.40 ± 0.54) for texture given by the panel of judges was recorded in T_2_ (40% Date paste, 30% Chick pea flour) followed by T_3_ (30% Date paste, 40% Chick pea flour) (7.00 ± 0.7) and minimum (4.80 ± 0.44) was recorded in T_4_ (20% Date paste, 50% Chick pea flour) as presented in Table 7. Mouth feel values in all four treatments were ranged between 4.40 ± 0.54 to 8.00 ± 0. Highest scores (8.00 ± 0.02) for mouth feel given by the panel of judges was recorded in T_2_ (40% Date paste, 30% Chick pea flour) followed by T_3_ (30% Date paste, 40% Chick pea flour) (7.40 ± 0.54) and minimum (4.40 ± 0.54) was recorded in T_1_ (50% Date paste, 20% Chick pea flour) as presented in Table 7. Overall acceptability was ranged from 5.20 ± 0.44 to 8.60 ± 0.54. Highest scores (8.60 ± 0.54) for overall acceptability given by the panel of judges was recorded in T_2_ (40% Date paste, 30% Chick pea flour) followed by T_3_ (30% Date paste, 40% Chick pea flour) (7.40 ± 0.54) and minimum (5.20 ± 0.44) was recorded in T_1_ (50% Date paste, 20% Chick pea flour) as presented in Table 7.

**Table 7:** Sensory attributes of different date bars

Treatment	Color	Flavor	Taste	Texture	Mouth feel	Overall
T _1_	6.40 ± 0.54c	5.80 ± 0.83c	4.40 ± 0.54d	5.40 ± 0.54c	4.40 ± 0.54b	5.20 ± 0.44c
T _2_	8.40 ± 0.54a	8.40 ± 0.54a	8.80 ± 0.44a	8.40 ± 0.54a	8.00 ± 0a	8.60 ± 0.54a
T_3_	7.60 ± 0.54b	7.40 ± 0.54b	7.60 ± 0.54b	7.00 ± 0.7b	7.40 ± 0.54a	7.40 ± 0.54b
T _4_	5.60 ± 0.54d	5.20 ± 0.44c	5.20 ± 0.44c	4.80 ± 0.44c	5.00 ± 0.7b	5.40 ± 0.54c

Different letters show significant variations (p ≤ 0.05) in columns; values are expressed as means ± standard deviation in ten independent determinations

T1: (50% Date paste, 20% Chick pea flour)

T2: (40% Date paste, 30% Chick pea flour)

T3: (30% Date paste, 40% Chick pea flour)

T4: (20% Date paste, 50% Chick pea flour)

Among all the four treatments T_2_ had the maximum score which contain 40% date paste and 30% chickpea flour. Yousif et al [62] reported that utilization of date paste as flour replacer in bread making showed excellent results regarding decrease in malt index and delaying gelatinization. They narrated that date paste addition in bread as partial replacer of wheat flour at 4% than 8–12% showed good physical and sensory attributes as well as total gas production and retention was improved at 4–6%. 4–8% addition of date paste has retarded the firming of crumb texture significantly. Manickavasagan et al [63] observed that utilization of date fruit as functional or substituted ingredient in many food preparations is of great importance as it could be ideal to use to substitute added sugars which only cause an in increase in calories without giving any health benefits.

## Conclusion

On the basis of above mentioned attributes this project was designed to explore the therapeutic and medicinal value of three Pakistani date varieties (Aseel, Hallawi and Khudravi) in comparison with one exotic date variety (Ajwa). Exotic Ajwa and indigenous Hallawi showed almost similar physico-chemical attributes like they both have very minimum amount of crude fiber and crude fat which make them suitable for hypercholesterolemic and hyperlipidemic patients. Lower levels of sodium were present in Ajwa, Hallawi and Khudravi which is beneficial for hypertensive patients. Aseel, Ajwa and Hallawi showed strong antioxidant potential and total phenolic contents. In bio-efficacy study, Ajwa and Hallawi showed maximum total cholesterol, LDL, triglyceride ALT, urea and creatinine lowering potential. According to the study it was concluded that Hallawi dates have the similar nutritional and therapeutic potential as Ajwa of being used as a cure of many metabolic disorders and date bar was prepared from Hallawi on the basis of bio-evaluation results. This study should also be done on humans for the validation of results. In nutshell, Hallawi could be potentially used for the treatment of hypercholesterolemic patients instead of Ajwa because it is very expensive date variety.

## Data Availability

The data generated for this study available in the manuscript.
